# Supplementary figures and images for: Genetic Landscape of Rare Autoinflammatory Disease Variants in Qatar and Middle Eastern Populations Through the Integration of Whole-Genome and Exome Datasets
Source: Front Genet. 2021 May 13;12:631340. doi: 10.3389/fgene.2021.631340 (PMC8155677; doi:10.3389/fgene.2021.631340)

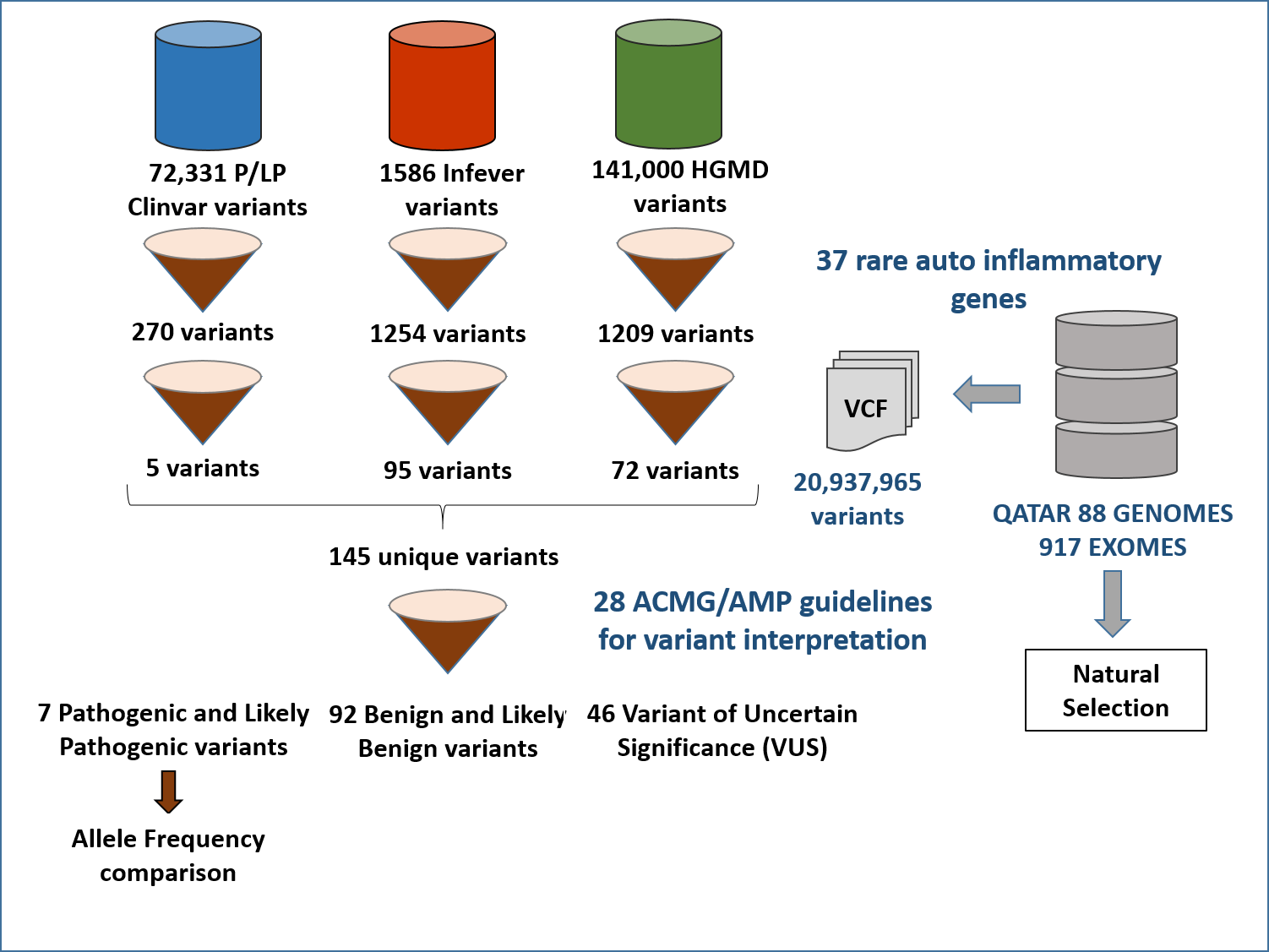

Supplement: Supplementary Figure 1 — Schematic summarizing the data analysis pipeline utilized for this study. [file Data_Sheet_1.zip › Image 1.TIF]
